# Cancer-associated fibroblasts in triple-negative breast cancer: heterogeneity, tumor-stroma crosstalk, and therapeutic resistance

**DOI:** 10.3389/fonc.2026.1977793

**Published:** 2026-10-13

**Authors:** Shijie Wei, Xiaoqing Xie, Yufeng Cao, Lingbo Wei

**Affiliations:** 1 Oncology Center III Department, Qingdao Hiser Hospital Affiliated of Qingdao University, Qingdao Traditional Chinese Medicine Hospital, Qingdao, China; 2 Oncology Center II Department, Qingdao Hiser Hospital Affiliated of Qingdao University, Qingdao Traditional Chinese Medicine Hospital, Qingdao, China; 3 Department of Geriatrics, Qingdao Hiser Hospital Affiliated of Qingdao University, Qingdao Traditional Chinese Medicine Hospital, Qingdao, China

**Keywords:** cancer-associated fibroblasts, extracellular matrix, immune exclusion, single-cell transcriptomics, therapy resistance, triple-negative breast cancer, tumor microenvironment

## Abstract

Triple-negative breast cancer (TNBC) comprises a clinically and biologically heterogeneous group of breast cancers defined by the absence of estrogen receptor and progesterone receptor expression and the lack of human epidermal growth factor receptor 2 (HER2) overexpression or amplification. Although systemic treatment has expanded beyond chemotherapy to include immune checkpoint blockade, poly(ADP-ribose) polymerase (PARP) inhibition in selected patients with germline BRCA1/2 variants, and antibody-drug conjugates in advanced disease, therapeutic resistance and disease recurrence remain major challenges. Cancer-associated fibroblasts (CAFs) are abundant stromal cells that contribute to the organization and function of the TNBC microenvironment. Rather than representing a single uniformly tumor-promoting population, CAFs encompass transcriptionally and functionally diverse states that vary across tumors, spatial niches, and treatment contexts. Current evidence supports roles for selected CAF populations in extracellular matrix (ECM) remodeling, paracrine signaling, metabolic exchange, immune-cell positioning, and adaptation to therapeutic stress. Recent single-cell and spatial studies have further identified ECM-rich, inflammatory, hypoxia-associated, and progenitor-like fibroblast states that correlate with distinct immune landscapes and treatment responses. However, many proposed CAF markers are shared across states, nomenclature remains inconsistent among datasets, and most CAF-directed therapeutic strategies are supported primarily by preclinical evidence. This review summarizes the current understanding of CAF heterogeneity in TNBC, examines the mechanisms through which fibroblast states influence tumor progression and antitumor immunity, and critically evaluates their contribution to chemotherapy and immunotherapy resistance. Particular attention is given to the distinction between mechanistic evidence and associative transcriptomic findings, as well as to the translational challenges that must be addressed before CAF-based biomarkers or therapies can be incorporated into routine TNBC management.

## 1 Introduction

Triple-negative breast cancer (TNBC) is defined by the absence of estrogen receptor and progesterone receptor expression and the lack of human epidermal growth factor receptor 2 (HER2) overexpression or amplification (1). This clinicopathologic definition encompasses several molecularly distinct tumor states rather than a single disease entity (2). TNBC is enriched for high-grade tumors and can show an increased risk of early recurrence and visceral metastasis, although clinical behavior varies substantially according to stage, tumor biology, host immunity, and response to systemic treatment (3, 4). The therapeutic landscape has also changed markedly. Cytotoxic chemotherapy remains a central component of treatment, but immune checkpoint blockade (ICB) has become established in selected early and advanced TNBC settings, adjuvant poly(ADP-ribose) polymerase (PARP) inhibition improves outcomes in eligible patients with germline BRCA1/2-associated high-risk HER2-negative disease, and antibody-drug conjugates have expanded treatment options for metastatic TNBC (5–9). These advances have made it increasingly important to understand biological determinants of both primary and acquired resistance.

The tumor microenvironment (TME) is one such determinant (4). TNBC develops within a multicellular ecosystem that includes immune cells, endothelial cells, adipocytes, extracellular matrix (ECM), and diverse mesenchymal populations (4, 10). Cancer-associated fibroblasts (CAFs) are a prominent stromal component in many TNBCs, but their abundance and phenotype vary between tumors and even between regions of the same tumor (11–13). CAFs can arise from tissue-resident fibroblasts and other mesenchymal precursors, and their state is shaped by tumor-derived signals, inflammatory mediators, hypoxia, mechanical stress, and treatment exposure (14–17). This plasticity is important because the biological effect of a fibroblast cannot be inferred reliably from a single marker such as alpha-smooth muscle actin (alpha-SMA) or fibroblast activation protein (FAP) (14, 18, 19).

Early work in breast cancer established that fibroblast populations differ in their expression of activation markers, secretory programs, and immunoregulatory properties (18). More recent single-cell RNA sequencing (scRNA-seq) and spatial profiling have revealed a broader continuum of myofibroblastic, inflammatory, ECM-producing, vascular-associated, antigen-presentation-like, and metabolically adapted fibroblast states (10–12, 20, 21). In TNBC, several studies have linked specific fibroblast programs to fibrosis, immune exclusion, angiogenesis, chemoresistance, or reduced response to ICB (20, 22–24). These observations have generated substantial interest in CAF-directed therapy, but they have also created a risk of overinterpreting dataset-specific clusters as universal biological subtypes (14).

A critical interpretation of the field therefore requires two distinctions. First, transcriptional association should be separated from functional causality. A CAF signature associated with poor outcome or treatment failure may identify a stromal state without demonstrating that the fibroblasts themselves are the dominant cause of resistance. Second, evidence obtained in breast cancer models should not automatically be treated as TNBC-specific. Several widely discussed stromal pathways, including neuregulin 1 (NRG1)-human epidermal growth factor receptor 3 (HER3) signaling and resistance to HER2-directed therapy, are biologically relevant in other breast cancer subtypes but cannot be generalized to TNBC without direct evidence (25, 26). This review focuses on mechanisms for which there is direct TNBC evidence whenever possible and explicitly treats emerging observations as provisional when independent validation is limited.

## 2 Literature search and selection

This narrative review was informed by a targeted literature search primarily conducted in PubMed/MEDLINE and supplemented by reference-list screening, targeted searches of journal and publisher databases, and clinical trial registries. The search was updated through September 2026 and used combinations of terms including “triple-negative breast cancer”, “cancer-associated fibroblast”, “CAF”, “tumor stroma”, “tumor microenvironment”, “single-cell RNA sequencing”, “spatial transcriptomics”, “extracellular matrix”, “immune exclusion”, “macrophage”, “chemotherapy resistance”, “immunotherapy resistance”, and related pathway-specific terms. Priority was given to studies using TNBC patient samples, TNBC-specific experimental models, single-cell or spatial profiling, and functional perturbation approaches. Evidence from mixed breast-cancer cohorts was included when directly relevant to CAF biology, whereas studies from other tumor types were used only for conceptual or translational context and are identified as such in the text. Recent studies were cross-checked for publication status and methodological details. Because this article is a narrative rather than a systematic review, no formal meta-analysis or risk-of-bias assessment was performed, and the literature selection may not be exhaustive. Accordingly, mechanistic, observational, and clinical findings are distinguished throughout the review wherever possible.

### 2.1 CAF heterogeneity and state plasticity in TNBC

The concept of CAF heterogeneity has moved the field away from a binary distinction between normal fibroblasts and activated fibroblasts (14, 15). In human breast tumors, CAF populations differ in their surface markers, transcriptional programs, spatial distribution, and capacity to influence immune cells (11, 18). A well-established breast cancer framework identified fibroblast subsets with distinct combinations of FAP, alpha-SMA, integrins, and other stromal markers, including a CAF-S1 population with prominent immunosuppressive properties (18). In TNBC, however, no single classification system has become universally accepted. Different studies use different tissue processing methods, sequencing platforms, clustering strategies, marker panels, and patient populations, leading to partially overlapping but nonidentical fibroblast states (10, 13, 14).

The most reproducible biological themes across datasets are more informative than the labels assigned to individual clusters. One recurring state is myofibroblastic, characterized by contractile and matrix-remodeling genes and often enriched for ACTA2, TAGLN, collagen-related genes, and transforming growth factor beta (TGF-beta)-responsive programs (10–12). A second recurring state is inflammatory or secretory, with increased expression of cytokines and chemokines such as C-X-C motif chemokine ligand 12 (CXCL12) and other immune-modulating mediators (11, 18). Additional states are enriched for ECM organization, vascular interaction, complement-associated programs, or stress responses (10, 12). These states should be viewed as context-dependent phenotypes along a continuum rather than immutable cell lineages (27, 28). Representative CAF states in TNBC, together with their defining features, evidence sources, functional validation, and current translational status, are summarized in **Table 1**.

**Table 1:** Representative CAF states and evidence hierarchy in triple-negative breast cancer.

CAF state or marker	Definition level	TNBC evidence source	Spatial context	Proposed biological role	Functional validation	Treatment/resistance phenotype	Human clinical evidence	Current translational status
Myofibroblastic CAFs (myCAF-like)	Transcriptionally and functionally defined state; commonly enriched for ACTA2, TAGLN, collagen-related and TGF-β-responsive programs	Breast-cancer single-cell datasets together with TNBC experimental and patient-derived studies	Predominantly ECM-rich and fibrotic stromal regions	Matrix deposition, contraction, tissue stiffening and mechanotransduction	Supported by multiple experimental models of TGF-β-dependent fibroblast activation and ECM remodeling	Associated with fibrosis, tumor-cell plasticity, impaired drug penetration and immune-cell access	Human breast-cancer and TNBC tissue studies support matrix-rich phenotypes, but no standardized myCAF biomarker is clinically validated	Biologically established program; not a clinically defined subtype
Inflammatory CAFs (iCAF-like)	Transcriptionally defined secretory/inflammatory state characterized by cytokine and chemokine programs	Human breast-cancer studies and TNBC single-cell analyses; BRCA1-mutated TNBC provides evidence of enhanced CAF–endothelial communication	Inflammatory and vascular-associated stromal niches; localization varies across tumors	Cytokine/chemokine signaling, immune-cell recruitment and vascular communication	Functional support exists for several secreted mediators, although some TNBC angiogenic interactions are based mainly on ligand–receptor inference	Linked to immune modulation and angiogenic programs; treatment relevance appears context dependent	Human TNBC evidence is predominantly observational/transcriptomic rather than interventional	Recurrent biological state, but not a uniform or stable CAF subtype
CAF-S1	Marker- and function-defined population characterized by FAP-high activated fibroblasts with additional surface-marker combinations	Human breast-cancer cohorts with preferential accumulation in TNBC and primary CAF functional assays	Stromal regions associated with regulatory T-cell accumulation	CXCL12-dependent T-cell recruitment/retention and additional immunoregulatory signaling	Strong functional evidence from primary human CAF–T-cell assays supports induction and maintenance of immunosuppressive T-cell phenotypes	Immunosuppression and resistance-associated immune phenotypes	Demonstrated in human breast-cancer specimens, including TNBC enrichment; no prospective CAF-S1-guided therapeutic trial	Well-characterized breast-cancer CAF framework; not equivalent to all iCAF states
ECM-rich CAFs (ecmCAFs)	Transcriptionally defined ECM-producing state with functional support	Integrated scRNA-seq from 18 TNBC patients, together with spatial and experimental analyses	Fibrotic, hypoxic regions; close spatial association with SPP1-positive macrophages	ECM deposition, stromal fibrosis and restriction of T-cell access; reciprocal stromal–myeloid signaling	Macrophage-derived TGFB1, IL1B and SPP1 were functionally linked to induction/maintenance of the ECM-producing fibroblast phenotype under hypoxia	Enriched in residual disease after neoadjuvant immunotherapy and associated with immune exclusion	Direct human TNBC evidence, but no prospective validation as a predictive biomarker	Relatively well-supported TNBC-specific state; still investigational for patient stratification
CA9-positive CAFs	Marker-defined hypoxia-associated CAF population with transcriptional and functional characterization	Multi-cohort TNBC single-cell analysis combined with spatial transcriptomics, multiplex imaging and fibroblast perturbation experiments	Hypoxic niches with close localization to SPP1-positive tumor-associated macrophages	Hypoxia-associated stromal–myeloid cooperation and immune suppression; VEGFA–NRP2-related communication has been implicated	CA9 perturbation and fibroblast–TNBC co-culture provide functional support; the complete VEGFA–NRP2 interaction remains incompletely validated causally	Associated with reduced effector T-cell infiltration and poorer immunotherapy response	Human multi-omics and spatial evidence; no prospective biomarker-guided trial	Emerging, multimodally supported TNBC state; clinical predictive value unvalidated
CD201-high (PROCR-high) CAFs	Marker-defined, progenitor-like transcriptional state	scRNA-seq from 10 TNBC patients, pseudotime analysis, cross-species analyses and external prognostic cohorts	Precise spatial niche has not been consistently established	Proposed early fibroblast state with progenitor-like, metabolic and immunoregulatory features	Direct functional validation of CD201 as the causal driver remains limited; pharmacologic studies have not established CAF-specific CD201 target engagement	Associated with immune-checkpoint programs and prognostic signatures; treatment implications remain preliminary	Human TNBC transcriptomic and prognostic associations without prospective clinical validation	Emerging research-defined state; not an established CAF subtype or therapeutic target
Myofibro-inflammatory CAFs (my_iCAFs)	Hybrid transcriptional state co-expressing myofibroblastic and inflammatory programs	TNBC-enriched state identified by integrated single-cell RNA and chromatin-accessibility analyses	Spatial organization has not yet been independently established across cohorts	Links contractile/ECM programs with inflammatory signaling, supporting a continuum between conventional myCAF- and iCAF-like phenotypes	Functional validation of the proposed regulatory program has been reported, but independent replication remains limited	Associated with tumor-promoting signaling programs; clinical treatment-response relevance remains unclear	No prospective human validation; current evidence is derived primarily from single-cell/multi-omic datasets and experimental validation	Very recent study-defined state; useful mainly as evidence against a rigid myCAF/iCAF dichotomy
Antigen-presentation-like CAFs (apCAF-like)	Transcriptionally defined state expressing antigen-presentation-associated programs	Public scRNA-seq data from nine TNBC patients integrated with bulk transcriptomic datasets	Spatial localization and interaction niches remain insufficiently characterized in TNBC	Proposed regulation of immune-cell communication, including CXCL13- and MIF-associated programs	Experimental confirmation is limited mainly to selected molecular features; direct antigen-presentation function in TNBC remains insufficiently established	Associated computationally with immune infiltration, prognosis and predicted immunotherapy response	Human transcriptomic associations only; no prospective predictive validation	Exploratory transcriptional state; functional and clinical significance requires further validation
Meflin-high / tumor-restraining CAF-like state	Marker-associated functional phenotype proposed to represent tumor-restraining fibroblasts	TNBC cell–fibroblast 3D co-culture, spontaneous TNBC mouse models and analysis of human TNBC tissue regions	Associated with vessel-rich stromal regions	Restriction of tumor-cell proliferation and modulation of stromal/vascular organization	Meflin overexpression in fibroblasts inhibited TNBC-cell growth in 3D culture; Meflin deficiency altered tumor vascular features in vivo	Potential tumor-restraining and tissue-supportive effects rather than a defined resistance phenotype	Human tissue observations are supportive but not prospective or therapeutic	Promising evidence that CAF functions are not uniformly tumor promoting; insufficient to establish a distinct clinical “rCAF” subtype

CAF labels shown here should be regarded as operational marker-defined populations, transcriptional states, or functionally supported phenotypes rather than immutable cell lineages. States identified in different datasets are not necessarily equivalent, and substantial overlap can occur between marker and transcriptional programs. “Human clinical evidence” in this table primarily denotes evidence obtained from patient-derived tissues or clinical cohorts and should not be interpreted as evidence of prospective therapeutic utility unless explicitly stated. None of the listed CAF states is currently validated for routine treatment selection in TNBC.

#### 2.1.1 CAF definition, ontogeny, and methodological considerations

CAFs are not defined by a single lineage marker. In most studies, they are identified operationally by their location within tumor stroma, mesenchymal morphology, exclusion of epithelial, endothelial, and hematopoietic lineages, and expression of combinations of fibroblast-associated markers such as FAP, platelet-derived growth factor receptors, alpha-SMA, or extracellular-matrix genes (14, 17). None of these markers is specific to all CAFs, and their expression can change with activation state and local microenvironmental signals (14). For this reason, marker-defined populations, transcriptionally defined states, and functionally validated fibroblast phenotypes should not be treated as equivalent categories.

CAF ontogeny is similarly heterogeneous. Local tissue-resident fibroblasts are considered an important source, but breast-cancer studies support additional contributions from perivascular stromal cells and fibroblast populations associated with the mammary fat pad (11, 17). Experimental studies have also shown that adipose-derived stromal cells can acquire CAF-like phenotypes after exposure to breast-cancer-derived signals, whereas bone-marrow-derived mesenchymal stromal cells can be recruited to mammary tumors and generate a functionally distinct fibroblast population (29, 30). Epithelial or endothelial contributions have also been proposed in cancer models, but their quantitative importance remains uncertain and should not be regarded as an established source of CAFs in TNBC (14, 17). Importantly, direct lineage-tracing evidence in human TNBC is limited. Current TNBC studies therefore describe fibroblast states more reliably than they define their developmental origins.

Single-cell technologies have greatly expanded this framework, but they also introduce technical limitations. Tissue dissociation can induce stress-related transcriptional programs and alter the relative recovery of stromal populations (31). Ambient RNA and doublets can further distort cell annotation or generate apparent hybrid expression profiles if they are not adequately controlled (32). In addition, dissociation-based scRNA-seq removes the original spatial relationships between fibroblasts and neighboring tumor, vascular, and immune cells. Spatial transcriptomic and proteomic studies partly address this limitation and have shown that fibroblast phenotypes are strongly shaped by their anatomical niches (21, 33). Accordingly, CAF clusters identified in different datasets should be interpreted as context-dependent states rather than fixed lineages. Their biological relevance is strongest when transcriptional findings are supported by spatial localization, protein-level confirmation, and functional perturbation.

#### 2.1.2 Myofibroblastic, inflammatory, and ECM-rich fibroblast states

Myofibroblastic and inflammatory programs recur across breast-cancer CAF datasets, although their boundaries are not fixed and individual fibroblasts may show overlapping features (11, 14, 15). Myofibroblastic CAFs are generally enriched for contractile and matrix-associated genes, including ACTA2, TAGLN, and collagen-related genes, and are commonly linked to ECM deposition, matrix contraction, and increased tissue stiffness (11, 14, 34). TGF-beta signaling is an important driver of myofibroblast activation, although it is not the only pathway involved (15, 35). In mammary fibroblast models, TGF-beta and CXCL12/SDF-1 signaling can help sustain a tumor-promoting myofibroblastic phenotype (35). TNBC-specific studies provide additional functional evidence. Tumor-derived Hedgehog signaling can reprogram CAFs toward an FGF5- and fibrillar-collagen-producing state that supports cancer-stem-cell plasticity and docetaxel resistance (22). Antifibrotic treatment with pirfenidone can also reduce collagen production and TGF-beta-associated stromal signaling in TNBC models, although meaningful antitumor activity was mainly observed when it was combined with doxorubicin (36). Importantly, alpha-SMA expression identifies only part of this biology. Some matrix-producing fibroblasts show limited alpha-SMA expression, whereas alpha-SMA-positive cells can have functions beyond matrix deposition (20). Thus, alpha-SMA should not be used alone to define a functionally uniform myofibroblastic CAF population.

Inflammatory CAF states are typically less contractile and more strongly enriched for secreted cytokines, chemokines, and growth factors (11, 18). CXCL12 is a recurrent mediator in fibroblast-immune communication and can influence immune-cell positioning through C-X-C motif chemokine receptor 4 (CXCR4) (18). Broader breast-cancer studies have linked fibroblast-derived CXCL12 to immune regulation, angiogenesis, and metastatic dissemination, but these findings should not be treated as direct TNBC-specific evidence (18, 37, 38). A study of BRCA1-mutated TNBC identified an inflammatory CAF population with enhanced outgoing angiogenic signals toward tumor endothelial cells, including vascular endothelial growth factor (VEGF)-related communication, illustrating how tumor genotype may shape the stromal compartment (23). Because much of the vascular interaction in this study was inferred from single-cell transcriptional and ligand-receptor analyses, the findings support an association between BRCA1-related inflammatory CAF states and angiogenic niches rather than a fully established causal vascular pathway (23). More broadly, inflammatory CAFs should be regarded as context-dependent secretory states rather than a uniformly protumorigenic fibroblast subtype.

ECM-rich fibroblast states have received particular attention in the immunotherapy setting. Recent single-cell analyses of TNBC treated with neoadjuvant immunotherapy identified ECM-producing CAFs enriched in residual disease (20). These fibroblasts were associated with a fibrotic microenvironment and reduced T-cell access to tumor regions (20). Spatial analyses further linked these fibroblasts to secreted phosphoprotein 1 (SPP1)-positive macrophages under hypoxic conditions, suggesting that fibrosis may be maintained through reciprocal stromal-myeloid signaling rather than by fibroblasts acting in isolation (20). Functional analyses further implicated macrophage-derived TGFB1, IL1B, and SPP1 in promoting or maintaining the ECM-producing fibroblast phenotype under hypoxic conditions (20). Together, these observations support a reciprocal stromal-myeloid circuit rather than a model in which fibroblasts act independently. However, ecmCAFs have not been prospectively validated as a predictive biomarker and are better regarded as a relatively well-supported TNBC fibroblast state than as a fixed clinical subtype.

#### 2.1.3 Emerging TNBC-associated fibroblast states

Recent single-cell, spatial, and multi-omic studies have proposed several additional CAF states with potential relevance to TNBC. However, these populations are supported by different levels of evidence and should not be regarded as equivalent or stable fibroblast lineages (14, 33).

A CD201-high fibroblast population, defined by high expression of the endothelial protein C receptor encoded by PROCR, was identified in single-cell data from TNBC and placed by pseudotime analysis near the early portion of a proposed fibroblast differentiation trajectory (39). This population was associated with distinct immune features and was used to derive a prognostic gene signature (39). These findings suggest that fibroblast differentiation state may add another dimension to stromal heterogeneity, but direct evidence that CD201 itself determines the biological behavior of these cells remains limited (39). At present, however, CD201-high CAFs should be considered an emerging research-defined state rather than a clinically established subtype, because the reproducibility of the phenotype and its therapeutic implications require validation across independent cohorts and experimental systems (39).

A second recent example is a carbonic anhydrase 9 (CA9)-positive CAF population identified in TNBC with features of hypoxia and immunotherapy resistance (24). This population was supported by integrated single-cell analyses, spatial profiling, multiplex imaging, and fibroblast perturbation experiments (24). CA9-positive CAFs were found in close proximity to SPP1-positive tumor-associated macrophages, and VEGFA–neuropilin 2 (NRP2)-related communication was implicated in this stromal-myeloid interaction (24). However, the complete VEGFA–NRP2 signaling pathway has not been established by direct functional perturbation at every step. A combined fibroblast-macrophage signature correlated with an immune-suppressed microenvironment and poorer treatment response (24). These data are mechanistically interesting, but the clinical value of CA9-positive CAFs as a predictive biomarker has not yet been established prospectively (24). Thus, CA9-positive CAFs are best regarded as an emerging but multimodally supported hypoxia-associated state rather than a clinically validated CAF subtype (20, 24).

Recent evidence also challenges a rigid separation between myofibroblastic and inflammatory CAFs. A 2026 single-cell RNA and chromatin-accessibility study identified a myofibro-inflammatory CAF state (my_iCAF) enriched in TNBC. These cells co-expressed myofibroblastic markers, including FAP and ACTA2, with the inflammatory marker CXCL12 (27). Multi-omic analyses implicated FOSB and a FOSB–HES1 regulatory program in this state, and experimental analyses supported its involvement in CAF-state switching (27). Because this observation is very recent and has not yet been independently reproduced across large spatially resolved TNBC cohorts, my_iCAFs are more useful at present as evidence of fibroblast-state continuity than as a new fixed CAF subtype (27).

Antigen-presentation-associated fibroblast programs have also been reported in TNBC. A 2025 study using public scRNA-seq data from nine TNBC patients identified an apCAF-like transcriptional population and linked it to MIF-associated intercellular communication and CXCL13 expression (40). However, most of the evidence was transcriptomic or computational, and experimental validation focused on selected molecular features rather than direct demonstration of antigen presentation by these fibroblasts in TNBC. The functional and predictive significance of apCAF-like states therefore remains exploratory (40).

Importantly, emerging CAF states are not necessarily tumor promoting. A recent TNBC study identified Meflin-expressing fibroblasts as a potential tumor-restraining phenotype. Meflin overexpression in fibroblasts reduced TNBC-cell proliferation in three-dimensional co-culture, whereas Meflin deficiency altered tumor vascular features in an autochthonous TNBC mouse model (41). Human TNBC tissues also contained Meflin-positive stromal cells (41). These findings provide direct TNBC evidence that fibroblast functions can include tumor-restraining or tissue-supportive effects. Nevertheless, they do not yet establish Meflin-high fibroblasts as a stable clinical “rCAF” subtype or a validated therapeutic biomarker (41). Taken together, these emerging populations illustrate why individual markers or dataset-specific cluster names should not be interpreted as fixed CAF lineages. Their biological significance is strongest when transcriptional classification is supported by spatial localization and functional validation, and their clinical relevance remains to be established prospectively (14, 33).

#### 2.1.4 CAF-related prognostic signatures: value and limitations

Bulk transcriptomic and single-cell datasets have generated numerous CAF-related prognostic scores in TNBC (13, 42–44). These approaches differ substantially in how CAF information is defined. Some classify tumors according to inferred CAF abundance or CAF-associated transcriptional patterns, whereas others derive multigene risk scores from CAF-enriched or single-cell-defined gene sets (13, 42, 43). For example, one study classified 335 TNBC samples into CAF-positive and CAF-negative groups and developed a nine-gene model associated with survival and immune features (42). Another study derived a CAF-related prognostic index using METABRIC and validated its prognostic performance in additional transcriptomic cohorts (43). More recent work has likewise combined single-cell CAF annotation with bulk TNBC datasets to construct multigene risk models, illustrating the growing use of CAF states as a source of candidate prognostic biomarkers (13, 44).

These models can reveal reproducible associations among stromal transcriptional programs, immune composition, and clinical outcome, particularly when performance is reproduced in independent datasets (42–44). However, external computational validation does not by itself establish clinical utility. Most existing CAF signatures were developed retrospectively from public transcriptomic cohorts rather than prospectively tested in biomarker-guided TNBC trials (42–44).

Their current clinical utility is nevertheless limited. Stromal signatures derived from bulk tissue can be influenced by tumor purity, immune-cell content, and the expression of nominal CAF genes by other cell types (14, 45). This is particularly important when a CAF state identified by single-cell analysis is subsequently represented by a gene signature in bulk tissue, because the resulting score may capture several cellular compartments rather than fibroblasts alone (33, 45). Machine-learning models may also perform less well when applied to cohorts that differ in platform, patient population, tumor stage, or treatment context. Most importantly, prognostic and predictive value should not be conflated. An association between a CAF score and survival describes prognosis, whereas a predictive biomarker should identify differential benefit from a specific treatment. Although some CAF signatures have been correlated with inferred or observed immunotherapy-response phenotypes, none has yet been prospectively validated to select TNBC patients for ICB or another CAF-directed treatment (42–44). A prognostic signature also does not demonstrate that the fibroblast population from which it was derived is itself a causal therapeutic target. For these reasons, CAF-based risk models should presently be regarded as investigational biomarkers rather than tools ready for routine treatment selection.

### 2.2 Mechanisms through which CAFs support TNBC progression

Available experimental and patient-derived evidence suggests that selected CAF states can modify TNBC biology through several interconnected processes, including soluble-factor signaling, extracellular-vesicle-mediated communication, matrix remodeling, and regulation of tumor-cell plasticity (14, 15). The strength of evidence is not uniform across these mechanisms. Some have been supported by direct perturbation in TNBC models, whereas others remain based mainly on transcriptional, spatial, or associative observations. These mechanisms are not independent. A stiffened matrix can alter growth-factor signaling, hypoxia can change the fibroblast secretome, and tumor-cell-derived signals can in turn reprogram fibroblast metabolism and phenotype (14, 15, 46). For example, TNBC-cell-derived ITGB4 can be transferred to fibroblasts and induce metabolic reprogramming, illustrating that stromal phenotypes may themselves be shaped by tumor-cell signals (46). The most informative model is therefore one of reciprocal tumor-stroma adaptation rather than unidirectional stimulation of cancer cells by CAFs.

#### 2.2.1 Paracrine growth-factor and cytokine signaling

Fibroblast-derived growth factors can alter receptor signaling in TNBC cells, although the level of mechanistic support differs among individual pathways. The HGF–MET axis is one of the better-established preclinical examples (**Figure 1**) (47, 48). Fibroblasts can provide HGF that activates MET in TNBC cells, enhances invasive behavior, and can confer resistance to epidermal growth factor receptor (EGFR) inhibition in experimental models (**Figure 1**) (48). A subsequent three-dimensional organotypic model using patient-derived CAFs further showed that stromal HGF activates MET and promotes invasion, epithelial-mesenchymal plasticity, and oncogenic signaling in TNBC cells (48). Together, these studies establish a functional stromal HGF–MET interaction in experimental TNBC systems. However, the EGFR-inhibitor resistance phenotype should be interpreted as a mechanistic model of stromal bypass signaling rather than as a clinically validated resistance mechanism in TNBC. Mueller et al. demonstrated the EGFR-resistance phenotype in TNBC cell-line models, whereas Singh et al. later confirmed HGF–MET signaling in a patient-derived organotypic system.

**Figure 1:**
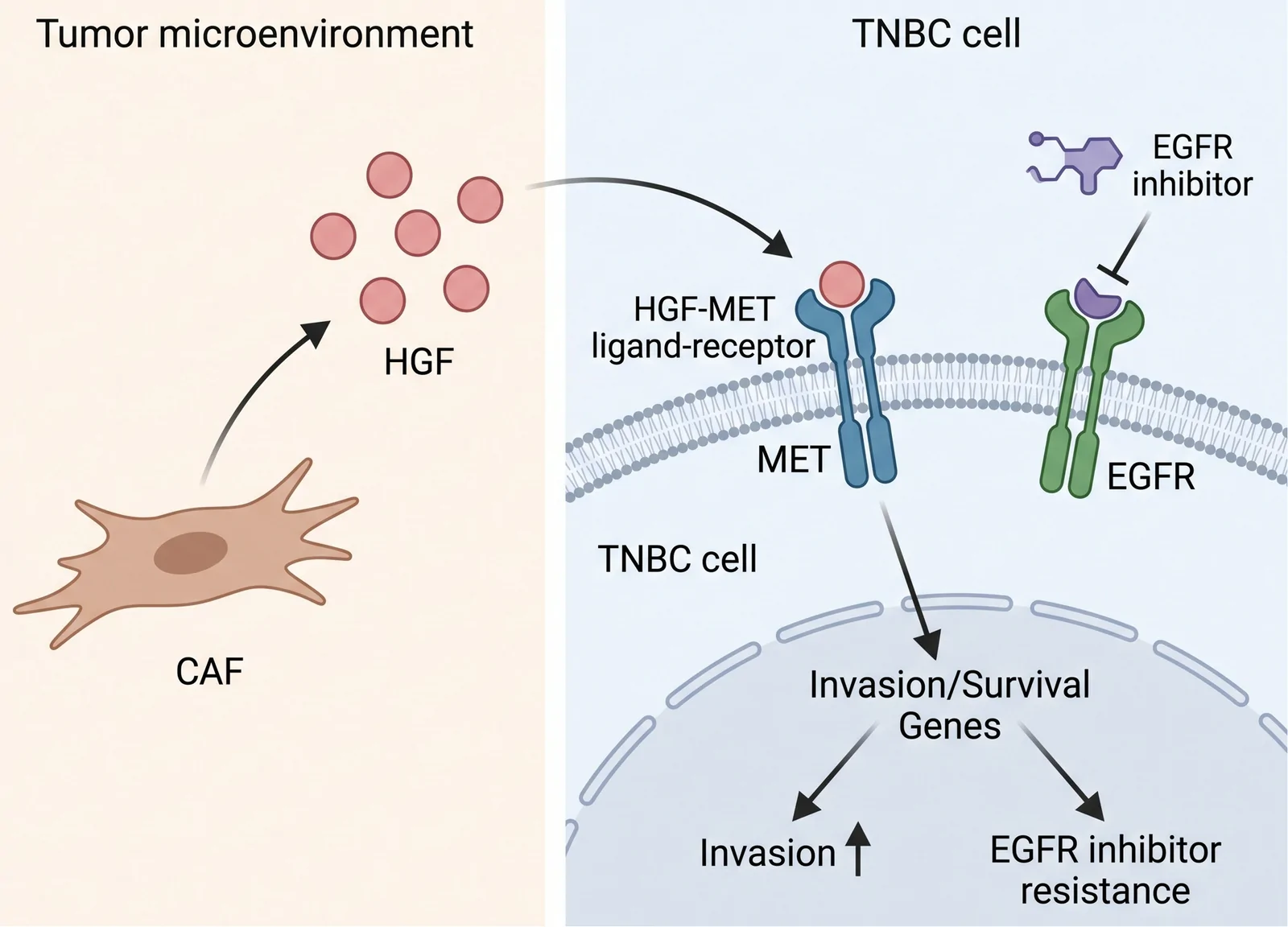
CAF-derived HGF–MET signaling promotes TNBC progression and resistance to EGFR inhibition. Cancer-associated fibroblasts (CAFs) secrete hepatocyte growth factor (HGF), which binds to and activates the MET receptor on triple-negative breast cancer (TNBC) cells. HGF–MET signaling promotes the expression of invasion- and survival-associated programs, thereby enhancing tumor-cell invasiveness and providing a bypass mechanism that reduces sensitivity to epidermal growth factor receptor (EGFR) inhibition.

NRG1 requires more cautious interpretation. Earlier mechanistic evidence for fibroblast-derived NRG1 came largely from luminal or HER2-positive breast cancer, where HER3-dependent signaling has been demonstrated directly (25, 49). In TNBC, a recent study of 174 patients reported increased NRG1 expression in tumor tissue and serum and found greater NRG1 secretion from CAFs than from normal fibroblasts (50). CAF-conditioned medium promoted TNBC-cell proliferation and migration, whereas NRG1 knockdown attenuated these effects. Xenograft experiments provided additional *in vivo* support for a tumor-promoting role of CAF-derived NRG1 (50). NRG1 is a ligand of HER3 and HER4, but receptor-specific dependence was not directly established in this TNBC study. The current evidence therefore supports CAF-derived NRG1 as a candidate paracrine mediator in TNBC, rather than a fully established NRG1–HER3/HER4 signaling mechanism.

TGF-beta is more difficult to assign to a single stromal mechanism because it acts in several cellular compartments. Broader CAF studies support its role in fibroblast activation, myofibroblastic differentiation, and ECM production (14, 15, 51). In TNBC tumor-cell models, TGF-beta1 and TGF-beta2 have been linked to epithelial-mesenchymal plasticity, invasion, and resistance to epirubicin (52, 53). These tumor-cell effects should not be interpreted automatically as CAF-derived signaling. Instead, TGF-beta is best viewed as a context-dependent regulator that can act on both stromal and malignant cells. Its biological consequence depends on the responding cell type, disease context, and interactions with other microenvironmental signals (14, 51). Other CAF-derived cytokines and chemokines may similarly influence angiogenesis and immune-cell behavior, but their relevance should be evaluated pathway by pathway rather than generalized to all TNBC CAFs (15).

#### 2.2.2 Extracellular vesicles and reciprocal tumor-stroma communication

Extracellular vesicles (EVs) provide a bidirectional route of communication between fibroblasts and TNBC cells (54–58). They can transfer proteins, lipids, and different classes of RNA between donor and recipient cells, thereby modifying cellular phenotypes beyond conventional ligand-receptor signaling (**Figure 2**) (54). Hypoxia can further alter both EV production and cargo, making EV-mediated signaling particularly relevant in hypoxic stromal niches (54, 55).

**Figure 2:**
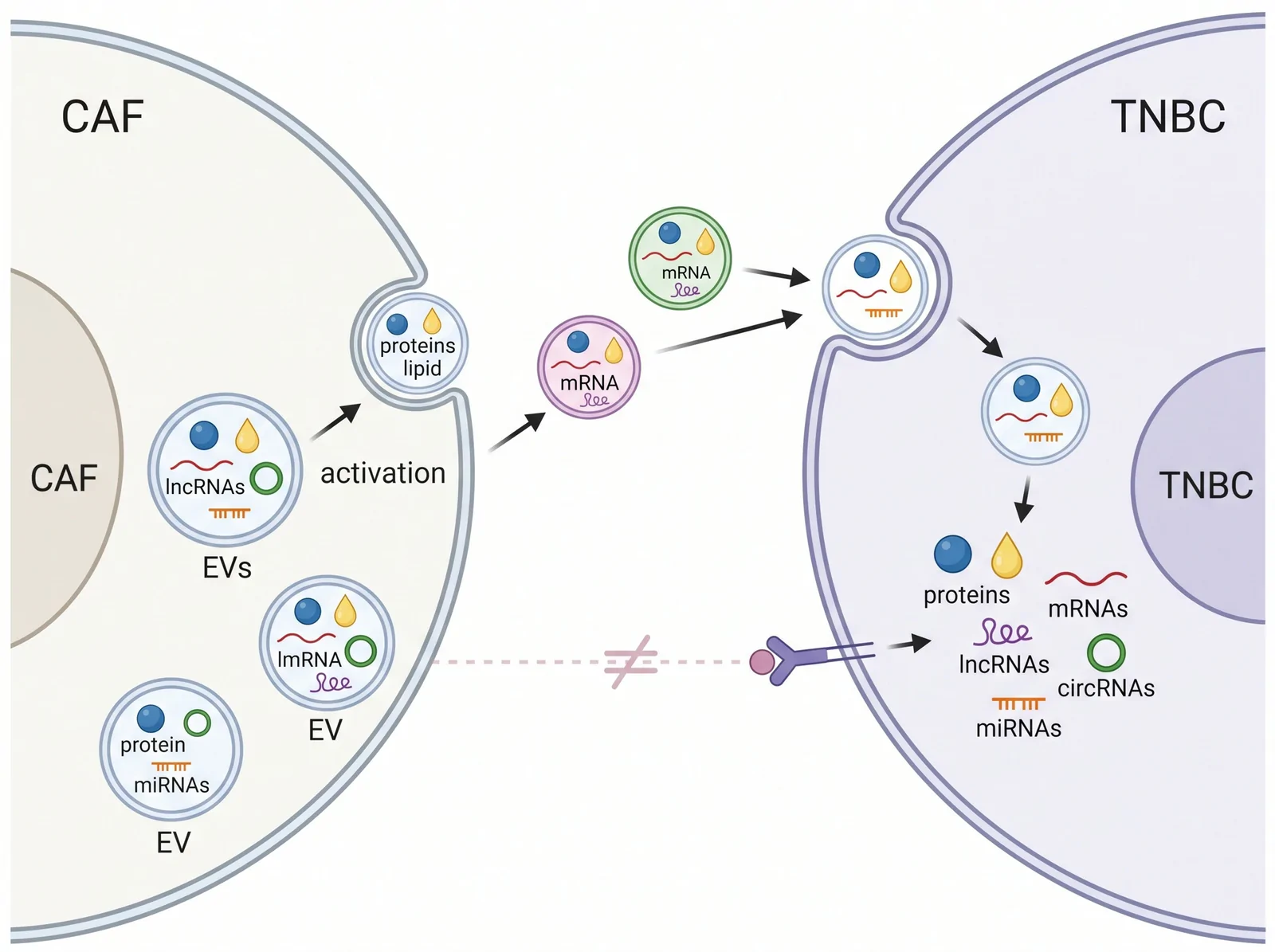
CAF-derived extracellular vesicles mediate tumor-stroma communication in TNBC. Cancer-associated fibroblasts (CAFs) release extracellular vesicles (EVs) that carry diverse bioactive cargoes, including proteins, lipids, mRNAs, long noncoding RNAs (lncRNAs), circular RNAs (circRNAs), and microRNAs (miRNAs). After uptake by triple-negative breast cancer (TNBC) cells, these vesicles deliver regulatory molecules that reshape tumor-cell signaling and phenotype, thereby supporting reciprocal tumor-stroma communication and contributing to malignant progression and treatment adaptation.

A recent TNBC study showed that hypoxic CAF-derived exosomal circSTAT3 was transferred to tumor cells and promoted stemness and cisplatin resistance through a miR-671-5p–NOTCH1 pathway (55). circSTAT3 depletion reduced these effects *in vitro* and in xenograft models (55). Elevated circSTAT3 was also detected in breast-cancer tissues and circulating EVs, although the clinical samples were not restricted to TNBC and did not establish CAFs as the source of circulating circSTAT3 (55). These results establish the pathway mainly at the experimental level, while the available human evidence remains associative.

Fibroblast-derived EVs can also influence chemotherapy response through other cargoes. SOD1-high fibroblasts were reported to release EVs enriched in miR-3960, which was transferred to TNBC cells and promoted cisplatin resistance by suppressing BRSK2-mediated phosphorylation of PIMREG and activating downstream NF-κB signaling (58). Experimental disruption of this pathway reduced the resistant phenotype, indicating that specific fibroblast-derived EV cargoes can contribute to drug adaptation in TNBC (58).

Communication also occurs in the opposite direction. TNBC-cell-derived EVs containing integrin subunit beta 2 (ITGB2) can promote fibroblast activation and subsequently enhance tumor growth, migration, and invasion (56). ITGB2 was also increased in TNBC tissues and serum EVs and was associated with more aggressive clinical features, although circulating EV measurements did not resolve their cellular origin (56). An earlier TNBC study showed that integrin subunit beta 4 (ITGB4) could be transferred from ITGB4-high tumor cells to fibroblasts through exosomes, where it promoted BNIP3L-dependent mitophagy and glycolytic reprogramming (46). The reprogrammed fibroblasts produced more lactate and in turn enhanced tumor-cell proliferation, epithelial-mesenchymal plasticity, and invasion (46). Together, these findings support a bidirectional EV-mediated feedback loop in which tumor cells can reshape fibroblast metabolism while fibroblast-derived signals modify tumor-cell behavior.

Broader breast-cancer studies have also linked stromal EV transfer to treatment adaptation, although these observations are not necessarily TNBC specific (57). Therapeutic targeting of EV release, uptake, or individual cargoes remains preclinical. The broad physiological roles of EVs, difficulty in defining the cellular origin of circulating vesicles, and lack of selective inhibitors currently limit clinical translation (54, 57).

#### 2.2.3 Epithelial-mesenchymal plasticity and cancer stem cell phenotypes

CAFs can support tumor-cell plasticity through soluble factors, extracellular vesicles, and matrix-derived cues (**Figure 3**) (14, 15, 22). In TNBC, epithelial-mesenchymal plasticity includes hybrid states rather than a simple switch between epithelial and mesenchymal phenotypes (59, 60). Hybrid phenotypes have been observed in mouse models, patient-derived xenografts, and archival TNBC samples (59). Several stromal signals can influence this plasticity. CAF-derived HGF can promote invasion and mesenchymal features in TNBC cells (48). Hedgehog-activated CAFs can also provide FGF5 and fibrillar collagen that support a cancer stem cell phenotype (22). TGF-beta can increase stem-like and mesenchymal features in TNBC models, although direct attribution of these effects to CAF-derived TGF-beta remains less certain (61). In broader breast cancer cohorts, CD10-positive GPR77-positive CAFs have been linked to cancer stem cell maintenance and chemoresistance (62). This finding supports a role for fibroblasts in stemness but is not specific to TNBC.

**Figure 3:**
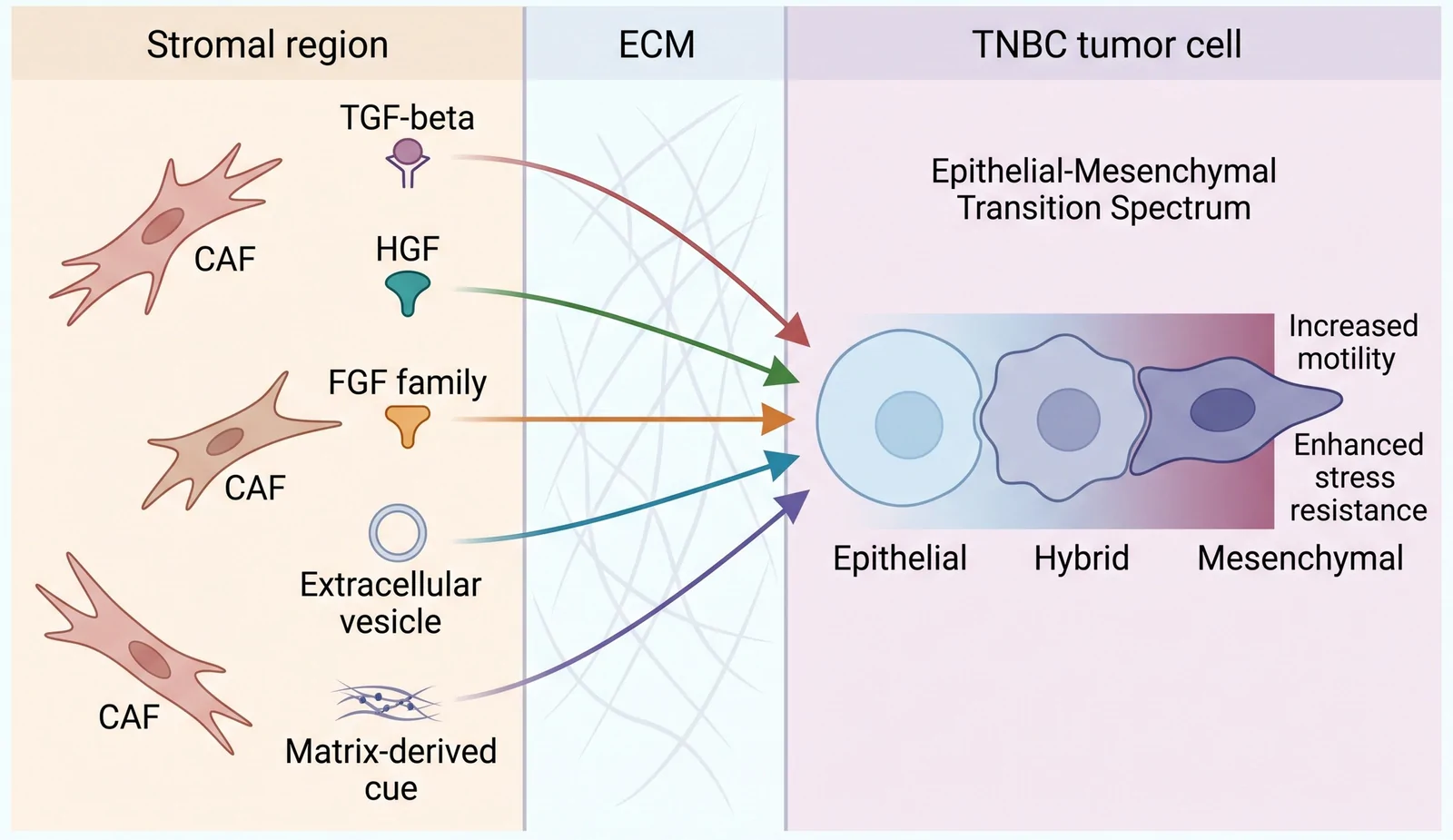
CAFs promote epithelial-mesenchymal plasticity in TNBC through soluble factors, extracellular vesicles, and matrix-derived cues. Cancer-associated fibroblasts (CAFs) influence triple-negative breast cancer (TNBC) cells through multiple stromal signals, including transforming growth factor-beta (TGF-beta), hepatocyte growth factor (HGF), fibroblast growth factor (FGF)-family signals, extracellular vesicles, and extracellular matrix (ECM)-derived cues. These inputs drive tumor cells along a spectrum of epithelial-mesenchymal transition states, from epithelial to hybrid and mesenchymal phenotypes, thereby increasing cellular motility and enhancing stress tolerance.

#### 2.2.4 ECM remodeling, stiffness, and invasion

The ECM is an active component of the TNBC stroma. CAFs deposit and remodel collagen, fibronectin, proteoglycans, and matrix-modifying enzymes (15, 20). This process changes matrix organization and tissue stiffness. In TNBC, ECM-rich CAFs have been linked to stromal fibrosis and reduced T cell access after neoadjuvant immunotherapy (20). Broader human breast cancer studies have also shown increased collagen deposition, linearization, and stiffness at invasive tumor regions. These features were prominent in basal-like tumors (63). A 2025 analysis of human TNBC samples further showed substantial variation in fibrillar collagen architecture between tumors (64). These findings indicate that ECM structure is a variable feature of TNBC rather than a uniform stromal property.

ECM mechanics can also alter fibroblast behavior. Patient-derived TNBC CAFs showed stiffness-dependent changes in invasion, and aryl hydrocarbon receptor activity contributed to these phenotypes (65). A recent TNBC study identified tumor-cell Jagged1 as another regulator of stromal remodeling (66). Jagged1 increased fibroblast activation, TGF-beta activity, collagen accumulation, and ECM alignment (66). Increased substrate stiffness also raised Jagged1 expression (66). These results support reciprocal signaling among TNBC cells, fibroblasts, and the surrounding matrix. Broader breast cancer models have shown similar feedback through YAP-dependent mechanotransduction in CAFs and periostin-integrin signaling in tumor cells (67, 68).

Fibrosis can influence treatment through both physical and signaling effects. In TNBC, ECM-rich CAFs have been associated with reduced T cell access to tumor regions (20). Pirfenidone reduced collagen accumulation and TGF-beta signaling in preclinical TNBC models (36). Its combination with doxorubicin improved tumor control and reduced metastatic burden, whereas pirfenidone alone had little antitumor activity (36). Broader breast cancer studies have shown that matrix stiffening can activate integrin and other mechanotransduction pathways that promote invasion (68, 69). ECM-targeted treatment may therefore alter both matrix structure and cell signaling. Direct evidence that improved drug penetration is the main mechanism remains limited in TNBC.

### 2.3 CAFs in the immunoregulatory microenvironment of TNBC

Immune activity in TNBC is highly heterogeneous. Some tumors contain abundant tumor-infiltrating lymphocytes, whereas others show limited immune infiltration (70, 71). Spatial analyses have also shown that immune cells may accumulate in stromal regions while remaining sparse within tumor-cell compartments (72). Selected CAF states can contribute to these immune patterns through chemokine signaling, ECM remodeling, vascular changes, and interactions with myeloid cells (18, 20, 23, 24). These effects can alter T-cell localization and myeloid-cell behavior. They can also influence vascular access and checkpoint-related immune responses. The extent of immune regulation differs among fibroblast states and treatment settings (18, 20, 24, 73).

#### 2.3.1 T-cell localization and immune exclusion

CXCL12-CXCR4 signaling can influence T-cell localization in breast tumors (**Figure 4**) (18, 74). In human breast cancer, CAF-S1 cells secreted CXCL12 and promoted the recruitment of CD4-positive CD25-positive T cells (18). CAF-S1 was enriched in TNBC, although the study included several breast cancer subtypes (18). In a murine 4T1 TNBC model, CXCR4 blockade with a liposomal AMD3100 formulation increased T-cell infiltration and improved the activity of anti-PD-L1 treatment (74). The intervention altered both stromal and immune compartments (74). These findings support a role for CXCL12-CXCR4 signaling in TNBC immune regulation, but they do not establish CAF-derived CXCL12 as the only source of this effect.

**Figure 4:**
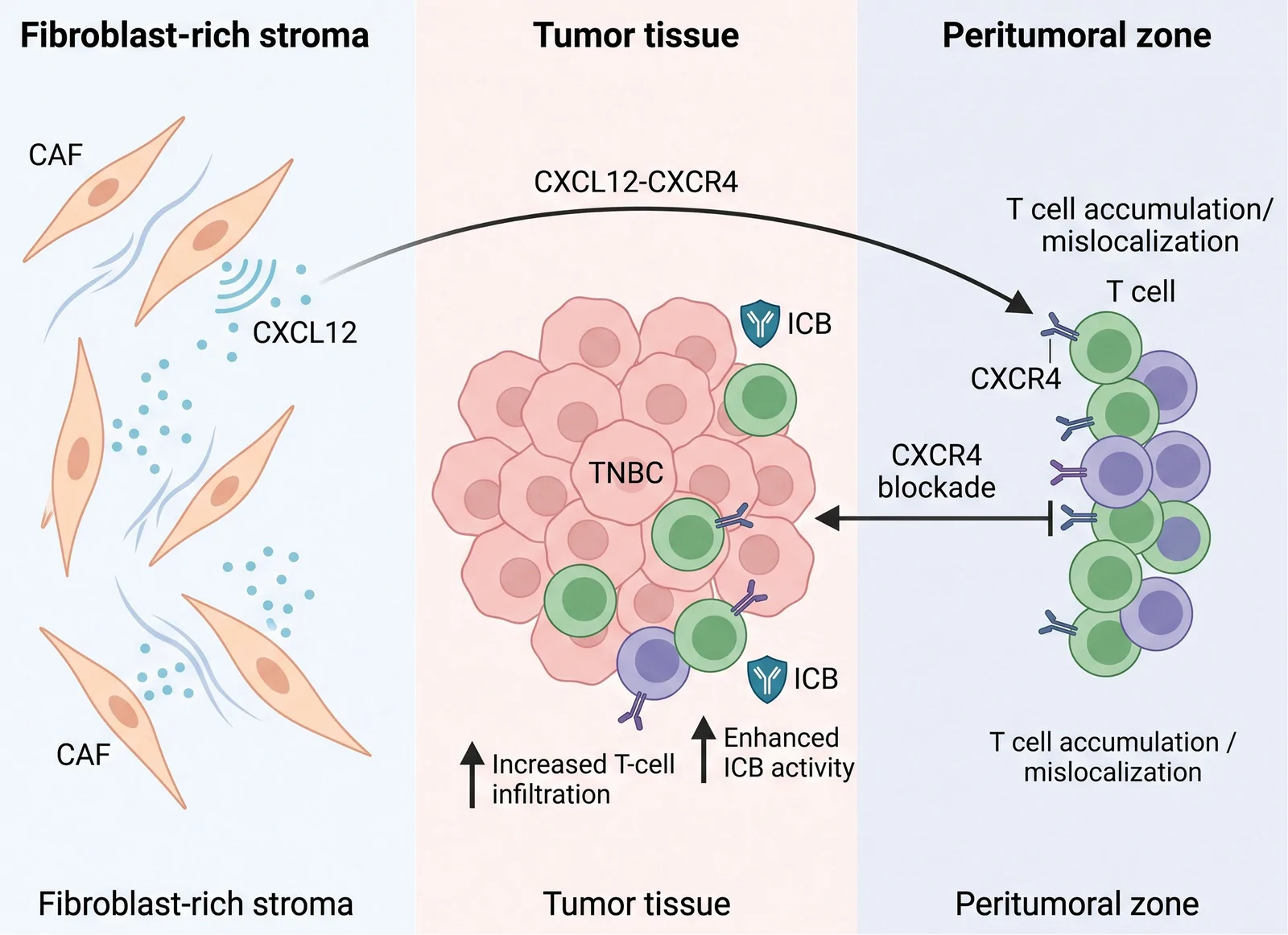
CAF-derived CXCL12 promotes T-cell exclusion in TNBC through CXCL12–CXCR4 signaling. Fibroblast-rich stroma produces C-X-C motif chemokine ligand 12 (CXCL12), which interacts with C-X-C motif chemokine receptor 4 (CXCR4) on T cells and restricts their effective infiltration into tumor tissue. Pharmacologic disruption of the CXCL12–CXCR4 axis can enhance intratumoral T-cell infiltration and improve the antitumor activity of immune checkpoint blockade (ICB), thereby promoting tumor-cell elimination.

ECM organization can also restrict T-cell access. In TNBC treated with neoadjuvant immunotherapy, ECM-rich CAFs were enriched in residual disease and were associated with stromal fibrosis and reduced T-cell access to tumor regions (20). Experimental breast cancer models provide additional functional support. CAF-rich tumors showed lower CD8-positive T-cell infiltration and reduced sensitivity to ICB (75). Genetic loss of Endo180 depleted a subset of alpha-SMA-positive CAFs and increased CD8-positive T-cell infiltration and ICB sensitivity (75). These findings support a role for selected CAF populations in immune exclusion, although direct clinical validation in TNBC remains limited.

Vascular abnormalities can provide another barrier to immune-cell entry (**Figure 5**) (76). In BRCA1-mutated TNBC, single-cell analysis identified inflammatory CAFs with increased CXCL and VEGF-related communication toward endothelial cells (**Figure 5**) (23). These fibroblast states were associated with angiogenic endothelial programs (23). Much of the proposed cellular communication was inferred from transcriptomic and ligand-receptor analyses. The study therefore links inflammatory CAFs with angiogenic niches but does not establish a direct causal pathway to T-cell exclusion. Broader cancer evidence shows that abnormal tumor vessels can impair immune-cell trafficking and reduce lymphocyte entry (**Figure 5**) (76).

**Figure 5:**
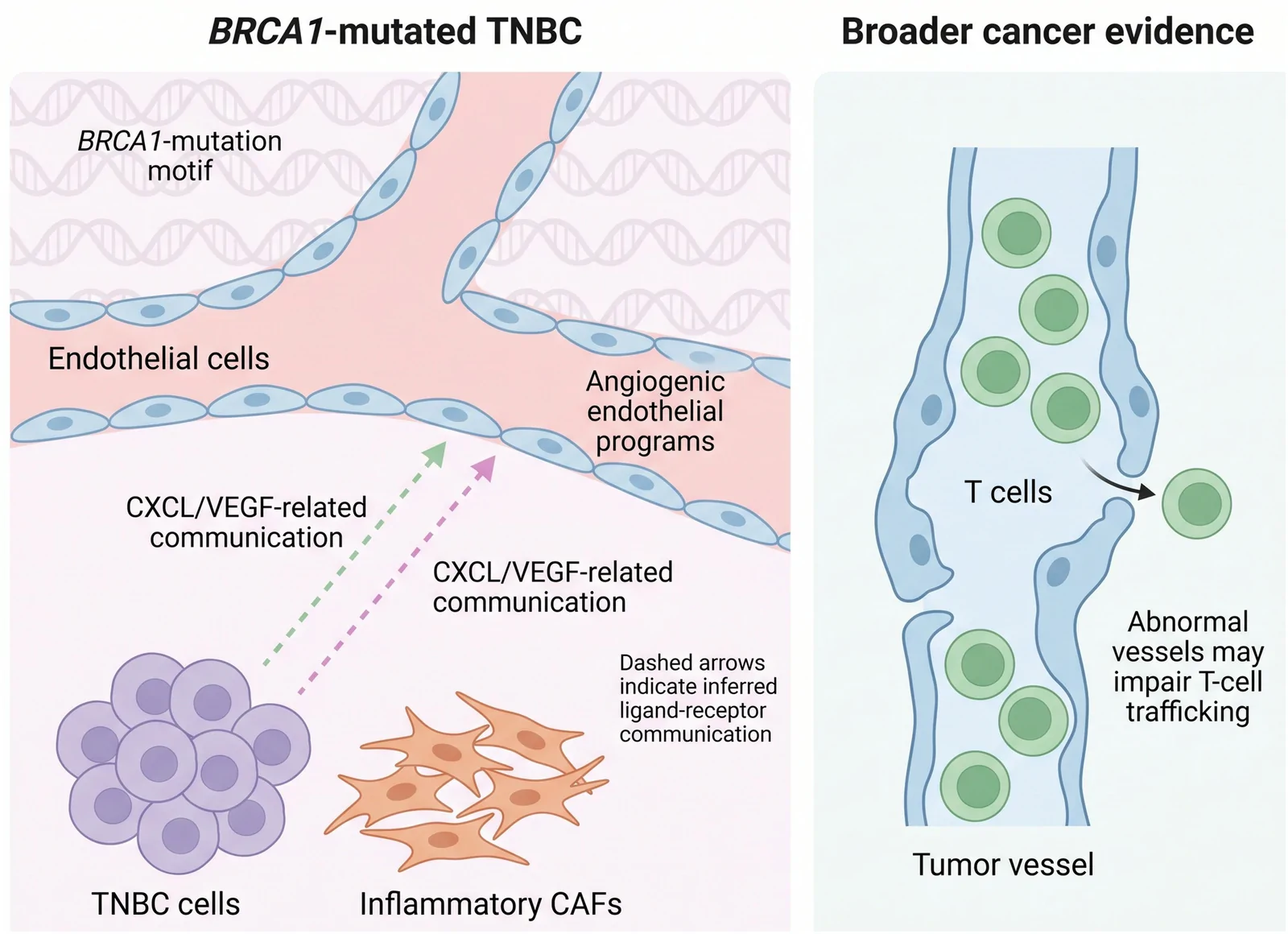
Inflammatory CAFs contribute to immune exclusion through stromal-vascular crosstalk in BRCA1-mutated TNBC. In BRCA1-mutated triple-negative breast cancer (TNBC), inflammatory cancer-associated fibroblasts (CAFs) promote angiogenic signaling toward endothelial cells and release angiogenic factors that increase vascular density and drive aberrant vascular remodeling. Although vessel density is increased, the resulting disorganized tumor vasculature impairs T-cell entry and trafficking, thereby contributing indirectly to immune dysfunction through the vascular compartment.

FGFR signaling has more direct preclinical support in TNBC (77). Human TNBC samples showed greater CAF accumulation in immune-excluded tumors, and FGFR1 was detected in alpha-SMA-positive stromal cells (77). In 4T1 and EMT6 syngeneic models, erdafitinib reduced CAF proliferation and migration. It also decreased VCAM-1 production through MAPK and ERK signaling (77). T-cell infiltration increased after FGFR blockade, and combination with ICB improved tumor control (77). These findings support CAF-dependent FGFR signaling as one mechanism of immune exclusion, but the therapeutic evidence remains preclinical.

#### 2.3.2 CAF-macrophage crosstalk

Fibroblasts and macrophages can regulate each other within the TNBC microenvironment (20, 24, 75). Several studies have identified distinct stromal and myeloid populations that participate in this interaction. A TNBC study identified a monocyte-derived STAB1-positive TREM2-high lipid-associated macrophage population with immunosuppressive activity (75). These macrophages were expanded in patients with resistance to ICB and suppressed T-cell activation and proliferation (75). Coculture experiments showed that TNBC-derived CAFs could reprogram blood monocytes toward this macrophage state (75). CAF-derived CXCL12 also promoted monocyte recruitment through CXCR4. Genetic depletion of the lipid-associated macrophage population reduced tumor growth in mouse models (75). These findings provide functional evidence that CAFs can influence both the recruitment and the state of myeloid cells in TNBC.

Reciprocal signaling in the opposite direction has also been described. ECM-rich CAFs were enriched in residual TNBC after neoadjuvant immunotherapy and were located close to SPP1-positive macrophages (20). Under hypoxic conditions, these macrophages produced higher levels of TGFB1, IL1B, and SPP1 (20). Functional experiments showed that SPP1-positive macrophages could promote the differentiation of normal breast fibroblasts toward an ECM-rich state (20). The resulting matrix remodeling and fibrosis were associated with reduced T-cell access to tumor regions (**Figure 6**) (20).

**Figure 6:**
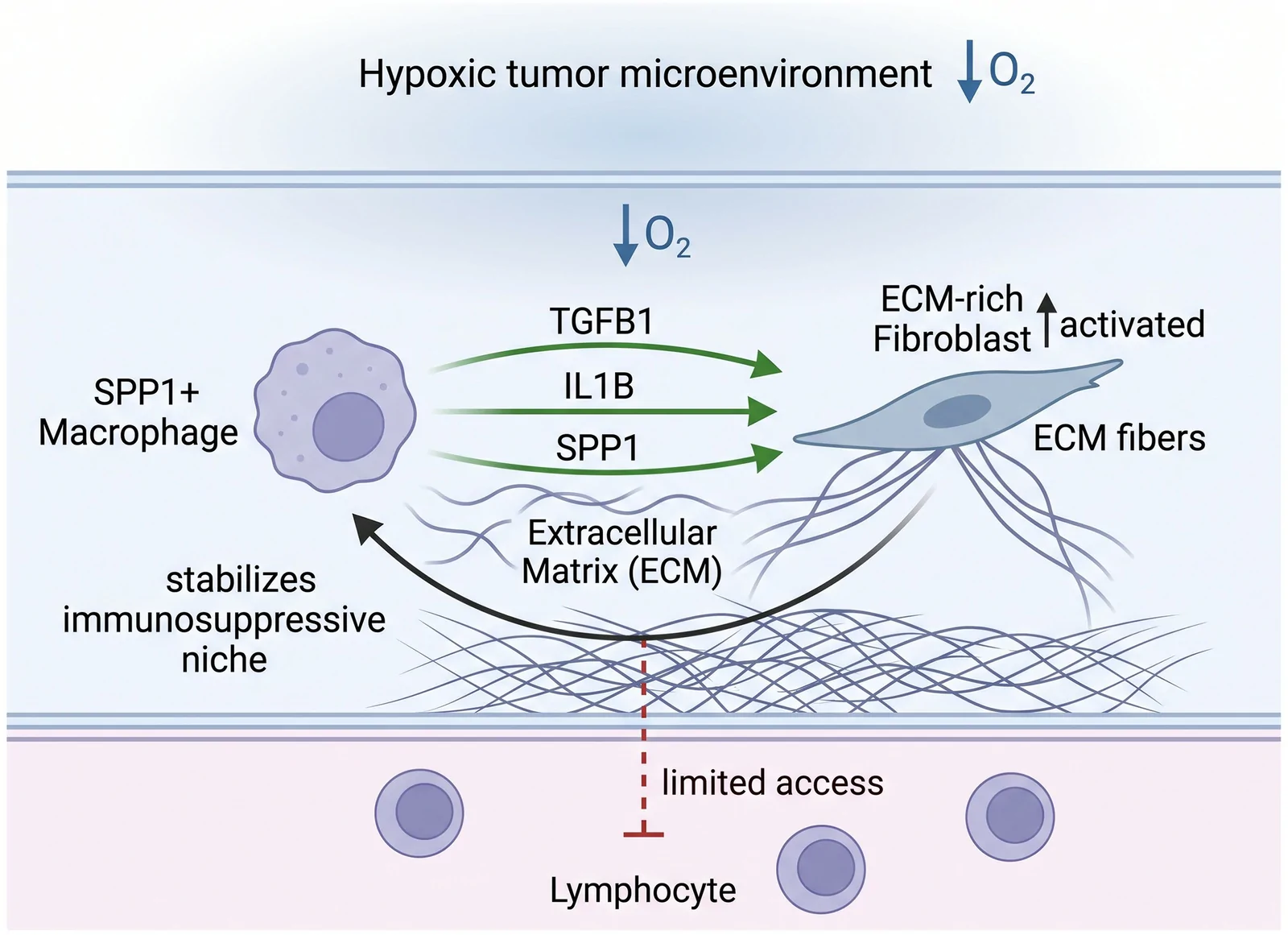
CAF–macrophage crosstalk promotes fibrosis and immune exclusion in the hypoxic TNBC microenvironment. Under hypoxic conditions, SPP1-positive macrophages provide signals including transforming growth factor beta 1 (TGFB1), interleukin 1 beta (IL1B), and SPP1 that promote or maintain an extracellular matrix (ECM)-rich fibroblast state. Activated fibroblasts enhance ECM deposition and fibrosis, which restrict lymphocyte access to tumor regions and contribute to the stabilization of an immunosuppressive stromal niche.

A second spatially defined stromal and myeloid niche involves CA9-positive CAFs and SPP1-positive tumor-associated macrophages (24). These populations were enriched in hypoxic TNBC regions and showed close spatial colocalization (24). Cell communication analyses implicated VEGFA and NRP2 signaling in their interaction. A high combined CA9-positive CAF and SPP1-positive macrophage profile was associated with lower effector T-cell infiltration and reduced response to immunotherapy (24). CA9 perturbation in fibroblasts altered TNBC-cell proliferation, migration, and invasion in coculture experiments (24). However, the specific contribution of VEGFA and NRP2 signaling to the stromal and myeloid interaction has not been fully established by direct perturbation. The combined profile has not yet been prospectively validated as a treatment-selection biomarker.

#### 2.3.3 Immune checkpoint signaling and antigen-presentation-associated programs

Programmed death-ligand 1 (PD-L1) can be detected on CAFs in a subset of TNBC specimens (78). In a retrospective surgical cohort of 61 patients, double immunofluorescence identified alpha-SMA-positive spindle cells that also expressed PD-L1 (78). CAF PD-L1 positivity measured with the 73–10 antibody was associated with longer overall survival. The same association was not observed with the SP142 or E1L3N antibodies. CAF PD-L1 positivity also correlated with PD-L1 expression in stromal tumor-infiltrating lymphocytes, but not with the stromal lymphocyte category used in the study (80). Interferon-related signaling was not assessed as an explanation for these findings. The cohort excluded patients who had received neoadjuvant chemotherapy, while adjuvant chemotherapy varied and was independently associated with overall survival (78). The study did not evaluate response to ICB. CAF PD-L1 therefore has prognostic rather than predictive evidence in this setting.

Fibroblasts can also regulate checkpoint pathways indirectly. In breast cancer, single-cell analysis identified CAF-S1 subclusters enriched for ECM or TGF-beta programs (79). These states were linked to primary resistance to immunotherapy in cross-cancer analyses (79). An ECM-rich CAF-S1 cluster increased PD-1 and CTLA-4 protein levels in regulatory T cells (79). A 2025 cross-cancer analysis that included TNBC identified a treatment-associated S100A4-high fibroblast program (80). *In vitro* experiments showed that fibroblast-derived S100A4 increased PD-L1 expression in tumor cells (80). These mechanisms indicate that fibroblasts can modify checkpoint signaling in neighboring cells. Their clinical importance in TNBC has not yet been established.

Antigen-presentation-associated fibroblast programs may provide another route of immune regulation. A 2025 analysis of public scRNA-seq data from nine TNBC patients identified an apCAF-like population with MIF-related communication and increased CXCL13 expression (40). Most of the evidence was transcriptomic or computational, and experimental validation focused on selected molecular features (40). Direct antigen presentation by these fibroblasts has not been demonstrated in TNBC.

### 2.4 CAF-mediated therapeutic resistance in TNBC

CAF-associated resistance mechanisms vary according to treatment and disease setting. Current evidence is strongest for cytotoxic chemotherapy and immune checkpoint blockade, whereas evidence involving PARP inhibitors and antibody-drug conjugates remains more limited.

#### 2.4.1 Resistance to cytotoxic chemotherapy

CAFs can alter chemotherapy response through matrix remodeling, paracrine signaling, and changes in tumor-cell state (22, 36, 81). In TNBC models, tumor-derived Hedgehog signaling reprogrammed CAFs to produce FGF5 and fibrillar collagen (22). This stromal program promoted a cancer stem cell phenotype and reduced sensitivity to docetaxel (22). Smoothened inhibition suppressed the CAF program in patient-derived xenografts and increased docetaxel sensitivity (22). These findings provide direct preclinical evidence that a defined tumor-to-CAF signaling pathway can modify chemotherapy response in TNBC.

Antifibrotic intervention provides additional experimental evidence. In TNBC models, pirfenidone reduced collagen production and TGF-beta-associated stromal signaling (36). *In vivo* treatment reduced fibrosis but did not substantially inhibit primary tumor growth or lung metastasis when used alone (36). Combination with doxorubicin produced greater inhibition of tumor growth and lung metastasis than either treatment alone (36). These results support stromal modulation as a chemotherapy-sensitizing strategy in this model rather than as an independent antitumor treatment.

Stromal effects on chemotherapy are not limited to direct protection from drug-induced cell death (82). In TNBC cell-line and stromal coculture models, fibroblast-derived factors did not consistently reduce acute chemotherapy sensitivity (82). Instead, stromal signals increased tumor-cell proliferation independently of treatment (82). Spatial analyses also associated proximity to stroma with increased tumor-cell proliferation *in vivo (*82). Mathematical modeling indicated that this proliferative advantage could accelerate recovery of residual tumor cells between treatment cycles and thereby reduce the probability of tumor elimination.

This mechanism differs from direct chemoprotection. It suggests that fibroblasts can influence treatment outcome by altering population recovery between chemotherapy cycles (82). Chemotherapy may also reshape stromal states through tissue injury and treatment-induced signaling (83). Longitudinal human TNBC studies remain limited, and the extent to which specific CAF states persist or emerge during standard chemotherapy has not been established.

#### 2.4.2 Ferroptosis resistance and metabolic adaptation

Lactate can function as both a metabolic substrate and a signaling molecule, and histone lysine lactylation provides a link between lactate availability and transcriptional regulation (84, 85). A 2025 study identified a CAF-dependent lactate pathway that altered ferroptosis and doxorubicin response in experimental TNBC (86). CAFs isolated from doxorubicin-resistant breast cancer tissues produced more lactate than CAFs from doxorubicin-sensitive tissues (86). Coculture with resistant CAFs increased histone H3 lysine 18 lactylation (H3K18la) and zinc finger protein 64 (ZFP64) expression in TNBC cells. Inhibition of lactate production reduced these effects and attenuated CAF-associated doxorubicin resistance (86).

ZFP64 provided a mechanistic link between histone lactylation and ferroptosis regulation. ZFP64 bound to the promoters of GTP cyclohydrolase 1 (GCH1) and ferritin heavy chain 1 (FTH1) and increased their transcription (86). ZFP64 silencing increased lipid peroxidation, intracellular ferrous iron, and other features of ferroptosis in doxorubicin-treated TNBC cells (86). It also increased sensitivity to doxorubicin (86). Overexpression of GCH1 or FTH1 partly reversed the effects of ZFP64 depletion. Mammary fat pad xenograft experiments provided additional *in vivo* support for this pathway (86).

The same study examined breast cancer tissues from 60 patients who had received doxorubicin-based chemotherapy before surgery (86). Thirty patients were classified as doxorubicin-resistant after recurrence within six months of treatment, whereas the other 30 were classified as doxorubicin-sensitive (86). Resistant tumors showed higher pan-histone lactylation, H3K18la, and ZFP64 expression. ZFP64 expression was also positively correlated with lactylation levels (86). However, the clinical cohort was described as breast carcinoma and was not clearly defined as an exclusively TNBC population. The human tissue findings therefore provide associative evidence rather than TNBC-specific clinical validation.

Together, these findings support a CAF-derived lactate–H3K18la–ZFP64 pathway that can suppress ferroptosis and reduce doxorubicin sensitivity in experimental TNBC (86). The causal evidence is derived mainly from cell and xenograft models, whereas the available human evidence remains correlative. The study did not establish H3K18la, ZFP64, GCH1, or FTH1 as predictive biomarkers for treatment selection, and therapeutic targeting of this stromal metabolic pathway remains experimental.

#### 2.4.3 Resistance to ICB

ICB is established in selected early and advanced TNBC settings (5, 6). However, CAF-specific evidence for resistance is more developed in early TNBC treated with neoadjuvant immunotherapy. In the GeparNuevo biomarker cohort, longitudinal gene-expression analysis included 247 tumor samples from 148 patients treated with chemotherapy with or without durvalumab (87). Stromal-related gene expression, including genes associated with collagen production, fibroblast activity, and angiogenesis, was associated with reduced pathological complete response and less favorable clinical outcome (87). Stromal gene expression also increased after durvalumab exposure. Because these measurements were derived from bulk tumor tissue, they support treatment-associated stromal adaptation but do not establish a CAF-specific causal mechanism.

More direct fibroblast evidence has emerged from single-cell and spatial studies. ECM-rich CAFs were enriched in residual TNBC after neoadjuvant immunotherapy and were associated with fibrosis and reduced T-cell access to tumor regions (20). SPP1-positive macrophages were spatially linked to these fibroblasts and could promote an ECM-rich fibroblast phenotype under hypoxic conditions (20). A separate study identified CA9-positive CAFs enriched in ICB nonresponders and closely colocalized with SPP1-positive macrophages (24). A high combined CA9-positive CAF and SPP1-positive macrophage profile was associated with reduced effector T-cell infiltration and attenuated immunotherapy response (24). These findings link fibroblast state and stromal-myeloid organization to ICB resistance, although neither signature has been prospectively validated for treatment selection.

Experimental models provide functional support for several stromal resistance mechanisms. In paired syngeneic mammary carcinoma models, CAF-rich tumors showed CD8-positive T-cell exclusion and resistance to combined CTLA4 and PD-L1 blockade (73). Genetic loss of Endo180 depleted a subset of alpha-SMA-positive CAFs, increased intratumoral CD8-positive T-cell infiltration, and improved sensitivity to ICB (73). In TNBC-specific models, CXCR4 blockade with liposomal AMD3100 remodeled stromal and immune compartments and improved the activity of anti-PD-L1 treatment in 4T1 tumors (74). FGFR blockade with erdafitinib also reduced CAF-associated barriers, increased T-cell infiltration, and enhanced ICB activity in immunocompetent TNBC models (77). These interventions support a functional contribution of stromal signaling to ICB resistance, but neither CXCR4 nor FGFR signaling is restricted to CAFs.

Together, current evidence supports several CAF-associated routes to ICB resistance, including T-cell exclusion, fibrotic matrix organization, myeloid reprogramming, and treatment-associated stromal adaptation (20, 24, 87). These mechanisms operate within a broader immune ecosystem and do not establish CAFs as the sole determinant of ICB response. Most CAF-specific human evidence currently comes from neoadjuvant early TNBC, whereas direct evidence in advanced or metastatic TNBC remains limited. No CAF state or stromal signature has yet been prospectively validated to select patients for ICB or for a CAF-directed combination strategy.

#### 2.4.4 Resistance to PARP inhibitors and antibody-drug conjugates

Compared with chemotherapy and ICB, direct evidence linking CAFs to PARP inhibitor resistance in TNBC remains limited. In patients with germline BRCA1 or BRCA2 pathogenic variants and high-risk HER2-negative early breast cancer, adjuvant olaparib improves clinical outcomes (7, 8). These trials did not evaluate CAF states as predictive biomarkers. A 2024 TNBC study identified a tumor-cell LAMB3-CXCL2 pathway associated with olaparib resistance and CAF infiltration (88). Genetic loss of LAMB3 increased olaparib sensitivity, whereas melatonin suppressed LAMB3 and CXCL2 expression, reduced CAF infiltration, and enhanced the antitumor activity of olaparib in experimental models (88). However, the study did not establish that a defined CAF population was required for olaparib resistance. The current evidence therefore links stromal changes to PARP inhibitor response at the preclinical level but does not establish a clinically validated CAF-mediated resistance mechanism.

Antibody-drug conjugates have become important in advanced TNBC. Sacituzumab govitecan targets TROP2 and delivers the topoisomerase 1 inhibitor SN-38. It improved progression-free and overall survival compared with single-agent chemotherapy in metastatic TNBC (9). Available mechanistic evidence for resistance has focused mainly on tumor-cell determinants rather than CAFs. Analysis of pretreatment and postprogression specimens identified absent TROP2 expression in a patient with primary resistance and acquired alterations affecting either TACSTD2/TROP2 or TOP1 in another patient (89). The TROP2 T256R alteration reduced plasma-membrane localization and antibody binding and conferred resistance to sacituzumab govitecan in experimental validation (89). These findings establish tumor-intrinsic routes of ADC resistance but do not demonstrate a CAF-mediated mechanism.

An emerging association between stromal state and ADC response has been suggested by recent single-cell and spatial analyses. A high CA9-positive CAF and SPP1-positive macrophage profile in TNBC was associated with an immunosuppressive microenvironment, and computational analyses suggested potential benefit from TROP2-, MUC1-, or NECTIN4-directed ADCs (24). This observation was not derived from prospective treatment selection in an ADC-treated TNBC cohort and does not establish the stromal signature as a predictive biomarker (24). Direct evidence that CAFs alter ADC delivery, payload sensitivity, or acquired ADC resistance in TNBC therefore remains insufficient.

Thus, CAF-related resistance to PARP inhibitors and ADCs is less well established than CAF involvement in chemotherapy or ICB resistance. PARP inhibitor evidence currently derives mainly from a single preclinical stromal association, whereas clinically documented resistance to sacituzumab govitecan is dominated by tumor-cell antigen and payload-target alterations. Prospective studies incorporating paired stromal profiling before treatment and at resistance will be required to determine whether specific CAF states have predictive or causal roles in these therapeutic settings.

#### 2.4.5 Evidence from human tumors and the need for causal validation

Human evidence supporting CAF-mediated therapeutic resistance in TNBC remains limited. Treatment-linked evidence is most developed in the neoadjuvant immunotherapy setting. In the GeparNuevo translational cohort, analysis of 247 tumor samples from 148 patients associated stromal programs with reduced pathological complete response and less favorable outcomes, although bulk transcriptomic data could not resolve individual CAF states (87). Single-cell and spatial studies have further linked ECM-rich CAFs and CA9-positive CAF–SPP1-positive macrophage niches to residual disease or reduced immunotherapy response (20, 24). However, these associations have not been prospectively validated for treatment selection. Interventional evidence remains sparse. The phase I EDALINE study evaluated sonidegib plus docetaxel in 12 patients with advanced TNBC and reported clinical benefit in three patients, including one complete response (22). The small uncontrolled cohort did not establish CAF-specific efficacy. No CAF state or CAF-derived signature has yet been prospectively validated as a treatment-selection biomarker. Future studies should incorporate predefined stromal biomarkers, paired tissue sampling, spatial analysis, and pharmacodynamic confirmation of stromal target engagement.

### 2.5 Therapeutic strategies targeting CAFs and the tumor stroma

CAF-directed therapy should account for fibroblast heterogeneity rather than assume that all activated fibroblasts are therapeutically harmful (14, 83, 90, 91). TNBC contains fibroblast states associated with tumor-promoting functions as well as emerging states with tumor-restraining features, including Meflin-expressing fibroblasts (41). Therapeutic approaches therefore increasingly focus on defined signaling pathways, fibroblast-state reprogramming, and targeted delivery rather than broad stromal depletion. Most CAF-directed strategies in TNBC remain preclinical or investigational. Their clinical development will require evidence that the relevant stromal state is present and that treatment modifies the intended CAF-dependent mechanism. Representative approaches, evidence contexts, biomarker hypotheses, translational barriers, and clinical status are summarized in **Table 2**.

**Table 2:** CAF- and stroma-directed therapeutic approaches in triple-negative breast cancer.

Target/pathway	Intervention/CAF strategy	Evidence/model context	Combination partner	Principal outcome	Biomarker hypothesis	Major translational barrier	Human clinical evidence	Current clinical status
TGF-β signaling	Pathway inhibition/antifibrotic reprogramming; reduces activated or fibrotic CAF programs rather than broadly depleting fibroblasts	TNBC co-culture, xenograft and stromal-remodeling models; supported by broader breast-cancer TGF-β biology	Chemotherapy or ICB	Reduced CAF activation and collagen-associated signaling can improve treatment sensitivity in selected models	TGF-β activity plus a fibrotic/ECM-rich CAF phenotype may identify pathway-dependent tumors	Redundancy, systemic toxicity, broad physiological roles and context-dependent tumor-suppressive effects	Early-phase TNBC TGF-β-containing regimens were not CAF-selected and have not established CAF-specific efficacy	Investigational; no validated CAF-directed TGF-β strategy in TNBC
CXCL12-CXCR4 axis	Functional blockade of CAF-associated chemokine signaling; does not require CAF depletion	Human CAF-S1 studies plus immunocompetent TNBC mouse models of CXCR4 blockade	ICB preclinically; eribulin in non-CAF-selected clinical testing	Preclinical blockade can increase T-cell infiltration and enhance checkpoint activity, but effects are context dependent	CXCL12-high fibroblast states together with CXCR4-expressing immune/stromal compartments	CXCR4 acts across immune, hematopoietic and tumor compartments; subtype dependence and lack of CAF-defined selection limit translation	No CAF-selected TNBC benefit demonstrated; a phase III HER2-negative metastatic breast-cancer study of CXCR4 inhibition plus eribulin was negative	Preclinical CAF rationale; not an established TNBC strategy
FGFR signaling in CAFs	Pathway blockade to suppress CAF-associated stromal signaling and partially reprogram an immune-excluding microenvironment	Immunocompetent TNBC mouse models treated with erdafitinib	ICB	Reduced stromal features, increased T-cell infiltration and improved antitumor activity preclinically	A CAF-associated FGFR signature may distinguish stromal dependence from tumor-cell FGFR signaling	On-target toxicity and pathway activity in both tumor cells and fibroblasts make CAF-specific attribution difficult	No prospective CAF-selected TNBC validation	Preclinical/investigational
Hedgehog-SMO stromal signaling	CAF-state reprogramming through Smoothened inhibition; suppresses a Hedgehog-induced FGF5/collagen program	TNBC models/PDXs followed by the small phase Ib EDALINE cohort	Docetaxel	Reduced stromal FGF5/collagen and cancer-stem-cell features; restored docetaxel sensitivity in models	An active tumor-to-CAF Hedgehog/FGF5-collagen program may define a dependent subgroup	Small clinical sample, no validated selection biomarker, pathway toxicity and no confirmatory trial	Phase Ib sonidegib plus docetaxel in 12 advanced/metastatic TNBC patients showed limited activity, including one complete response	Early clinical proof of principle; not standard care
ECM/fibrosis modulation	Antifibrotic stromal modulation; reduces collagen production/fibrotic signaling without selective CAF depletion	TNBC preclinical studies, including pirfenidone-based remodeling	Doxorubicin or other cytotoxic therapy	Pirfenidone alone was weak but improved chemotherapy response and reduced metastatic burden in models	Collagen architecture, stiffness and ECM-rich CAF signatures may define fibrotic dependency	ECM heterogeneity, incomplete CAF specificity, normal tissue repair effects and uncertain mechanism of benefit	No TNBC clinical trial has established CAF-directed antifibrotic efficacy	Preclinical; best viewed as a sensitizing concept
FAP-directed targeting	Targeted depletion, immune engagement or drug delivery to FAP-expressing activated fibroblasts	TNBC nanoparticle/cell-therapy models; FAP-directed agents also in early pan-solid-tumor development	Chemotherapy, ICB or engineered immune-cell approaches	Preclinical TNBC studies can reduce fibroblast-rich stroma, enhance immune access and improve tumor control	High stromal FAP plus spatial confirmation of the relevant fibroblast compartment	FAP is also expressed by nonmalignant activated fibroblasts; wound-healing functions, heterogeneity and off-tumor effects are concerns	Human FAP-targeted agents are in early pan-solid-tumor trials, but CAF-defined TNBC efficacy is unproven	Clinical translation outside TNBC; no validated TNBC indication
CAF lactate-histone lactylation-ferroptosis axis	Metabolic/epigenetic disruption to reverse a CAF-supported drug-tolerant state rather than eliminate fibroblasts	TNBC co-culture/cell systems, xenografts and human tumor-tissue association studies	Doxorubicin; conceptually ferroptosis-inducing approaches	Disrupting the CAF-lactate-H3K18la-ZFP64-GCH1/FTH1 pathway restored ferroptotic and chemotherapy sensitivity experimentally	CAF/lactate features plus H3K18la, ZFP64 and ferroptosis-protective markers may identify pathway-active tumors	Lactate metabolism is systemic, ferroptosis pathways are redundant, and the intact causal pathway lacks prospective human validation	Human samples support association of H3K18la/ZFP64 with doxorubicin resistance; no therapeutic validation	Mechanistically compelling but preclinical
CAF-state reprogramming (AhR- or TGF-β/SMAD-related)	Reprogramming invasive/activated CAF states while preserving nonmalignant stromal functions	Patient-derived TNBC CAF mechanobiology and preclinical models, including Huaier-mediated CAF suppression	PD-1 blockade in selected models	Modulating fibroblast regulators can reduce invasive/activated stromal phenotypes and sometimes improve immunotherapy response	AhR-dependent invasive features or TGF-β/SMAD-active CAF states are exploratory selection markers	CAF plasticity, uncertain pharmacologic specificity, heterogeneous targets and limited clinical pharmacology	Patient-derived CAF data support relevance, but no reprogramming regimen has shown TNBC clinical efficacy	Experimental/preclinical
CAF-directed nanomedicine and multifunctional delivery	Platform-dependent CAF targeting/depletion/reprogramming with localized cytotoxic, metabolic or immunomodulatory payloads	TNBC mouse models using FAP nanoparticles, relaxin formulations, nanomicelles and precision CAF nanoregulators	Chemotherapy, ICB or CAR-T, depending on platform	Preclinical platforms can reduce collagen/CAF abundance, increase CD8-positive T-cell infiltration and improve chemo-immunotherapy	FAP-high/fibrotic stroma, ECM density and payload-specific pathway activity may guide selection	Manufacturing reproducibility, biodistribution, target specificity, immunogenicity, scale-up, safety and interspecies differences	No TNBC clinical efficacy data for the CAF-directed nanoplatforms reviewed here	Preclinical proof of concept; substantial translation remains

Human evidence is distinguished from preclinical evidence and does not imply CAF-specific efficacy unless explicitly stated. None of the approaches listed is currently an established CAF-directed standard treatment for TNBC.

#### 2.5.1 Targeting stromal signaling and fibroblast activation

TGF-beta is an important regulator of fibroblast activation, matrix deposition, immune regulation, and tumor-cell plasticity, but it is not a CAF-specific target (15, 83, 90). TGF-beta signaling acts in malignant, immune, endothelial, and stromal compartments, and its biological effects vary according to cellular and disease context. Broad pathway inhibition may therefore affect normal tissue homeostasis and tumor-suppressive functions in addition to tumor-promoting stromal activity (83, 90). Parallel signaling pathways can also maintain fibrotic or immunosuppressive phenotypes when TGF-beta signaling is inhibited, which complicates patient selection and pharmacodynamic assessment. Clinical experience also illustrates these limitations. In a phase 1 expansion cohort, 33 patients with previously treated advanced TNBC received bintrafusp alfa, a bifunctional TGF-beta trap and PD-L1 inhibitor (92). The objective response rate was 9.1%, median progression-free survival was 1.3 months, and median overall survival was 7.7 months (92). HMGA2 emerged as a candidate response biomarker, but patients were not selected according to CAF state or stromal TGF-beta activity (92). The study therefore demonstrates clinical feasibility of combined TGF-beta and PD-L1 blockade but does not establish CAF-directed efficacy.

FGFR inhibition provides another experimental strategy. Erdafitinib reduced CAF proliferation and migration, decreased VCAM-1 production, increased T-cell infiltration, and enhanced ICB activity in immunocompetent TNBC models (77). However, FGFR signaling is not restricted to fibroblasts, and systemic FGFR inhibition can also affect malignant and normal tissues. Clinical FGFR inhibitors are associated with class-specific toxicities including hyperphosphatemia and ocular adverse events (93). The antitumor effects of FGFR inhibition therefore cannot be attributed to CAF modulation without stromal target-engagement data. Clinical development in TNBC would require biomarkers that identify tumors with a relevant CAF-associated FGFR program. CXCL12-CXCR4 blockade has also increased T-cell infiltration and enhanced anti-PD-L1 activity in the 4T1 TNBC model (74). However, the intervention altered both stromal and immune compartments, and CXCR4 signaling is not restricted to CAFs (74). Therapeutic effects therefore cannot be assigned specifically to CAF-derived CXCL12. Clinical translation will require biomarkers that identify tumors with active CXCL12-CXCR4 signaling and clarify the cellular compartments responsible for the relevant treatment response (90).

Hedgehog pathway inhibition provides one of the earliest examples of stromal targeting reaching a TNBC clinical cohort (22). Preclinical studies showed that Smoothened inhibition suppressed a Hedgehog-induced CAF program and restored docetaxel sensitivity (22). In a small phase I study, sonidegib combined with docetaxel produced clinical benefit in a subset of patients, but the cohort was too small to establish efficacy and the approach has not become standard care (22). Its lasting value is conceptual: stromal pathway inhibition may work best in biomarker-defined tumors in which a specific tumor-to-CAF circuit is active (22, 90).

#### 2.5.2 Reprogramming rather than eliminating CAFs

Because CAF states are plastic, state reprogramming provides an alternative to broad fibroblast depletion (83, 90). In patient-derived TNBC CAFs, invasive behavior was influenced by matrix stiffness and aryl hydrocarbon receptor (AhR) activity (65). AhR upregulation increased CAF invasion, whereas AhR suppression reduced invasion across mechanically distinct fibroblast populations (65). These findings identify AhR as a candidate regulator of CAF behavior, although therapeutic efficacy has not been demonstrated *in vivo* or clinically.

A second example is Huaier, which reduced myofibroblastic CAF differentiation through TGF-beta-SMAD signaling and enhanced PD-1 blockade in TNBC mouse models (94). These data support the feasibility of modifying CAF state rather than eliminating fibroblasts, but the evidence remains preclinical and no CAF-reprogramming strategy has been clinically validated in TNBC (94).

#### 2.5.3 Nanomedicine and CAF-directed delivery

Nanomedicine has been used to increase stromal drug delivery or combine CAF modulation with chemotherapy and immunotherapy (95–97). FAP-targeted nanoparticles, biomimetic particles, relaxin-containing formulations, and multifunctional nanomicelles have shown the ability to alter collagen deposition, CXCL12-related signaling, or fibroblast abundance in TNBC models (95–98). Several systems have increased intratumoral cluster of differentiation 8 (CD8)-positive T-cell infiltration and improved tumor control when combined with cytotoxic drugs or ICB (95–97).

The strength of this evidence is predominantly preclinical (95–97). Nanoparticle biodistribution, stromal target specificity, reproducibility, immunogenicity, manufacturing complexity, scale-up, long-term safety, and interspecies differences remain important barriers to clinical translation (83, 90). In addition, FAP is not exclusive to malignant fibroblasts and can be expressed in other activated stromal contexts (99). Therefore, current nanodelivery studies should be described as proof-of-concept platforms rather than near-term clinical solutions.

A recent precision CAF nanoregulator study in TNBC further supports the possibility of targeting fibroblast-rich stroma while simultaneously improving chemo-immunotherapy (97). Such work is technically sophisticated and biologically informative, but its therapeutic relevance will depend on reproducibility, scalable formulation, safety, and demonstration of benefit in human trials (97).

#### 2.5.4 Biomarkers and clinical translation

A central translational challenge is identifying patients with a biologically relevant and therapeutically actionable CAF state (33, 90). Because single markers do not capture CAF heterogeneity, composite biomarkers that integrate fibroblast state, spatial organization, matrix features, and interacting immune populations may be more informative (14, 20, 24, 33). Recent TNBC studies of ECM-rich CAFs and CA9-positive CAF-SPP1-positive macrophage niches support this approach, although these signatures have not been prospectively validated for treatment selection (20, 24).

scRNA-seq and spatial transcriptomics are valuable discovery tools but are not yet practical as routine clinical assays (100, 101). Translation will require simplified approaches such as immunohistochemistry, multiplex imaging, targeted RNA panels, or digital pathology, followed by prospective validation linked to a specific treatment decision (33, 90).

Future trials should distinguish CAF-directed effects from secondary stromal changes. Paired pretreatment and on-treatment tissue, spatial immune analysis, and pathway-specific pharmacodynamic markers will be needed to determine whether stromal target engagement contributes to therapeutic benefit.

## 3 Conclusion

CAFs are an important but heterogeneous component of the TME in TNBC. This heterogeneity includes tumor-promoting states as well as emerging fibroblast states with potential tumor-restraining functions. The most consistent evidence indicates that selected fibroblast states contribute to ECM remodeling, paracrine growth-factor signaling, tumor-cell plasticity, immune-cell positioning, myeloid crosstalk, and adaptation to therapeutic stress. Recent single-cell and spatial studies have refined this framework by identifying ECM-rich, hypoxia-associated, inflammatory, and progenitor-like fibroblast states and by showing that their biological effects depend on spatial relationships with tumor cells, endothelial cells, and immune populations (20, 23, 39).

The field should nevertheless avoid treating every transcriptomic cluster as a stable CAF subtype or every association with poor outcome as evidence of causality. Several recent TNBC findings, including CA9-positive CAF-SPP1-positive macrophage interactions, CD201-high fibroblast states, exosomal circSTAT3 signaling, and CAF-associated histone lactylation and ferroptosis resistance, are recent and require independent validation (24, 39, 55, 86). They expand the mechanistic landscape but do not yet define clinically established biomarkers or therapeutic targets.

CAF-directed therapy remains an experimental strategy. Preclinical data support modulation of TGF-beta-associated fibrosis, FGFR signaling, CXCL12-CXCR4 communication, Hedgehog-dependent stromal programs, fibroblast metabolism, and CAF-targeted nanodelivery (22, 36, 74, 77). Limited early clinical studies have evaluated stromal pathway targeting, but no CAF-specific intervention is currently part of standard TNBC management. Clinical translation will therefore require biomarker-guided modulation of defined tumor-stroma dependencies rather than indiscriminate fibroblast depletion. Integrating spatial biology with functional validation and prospective clinical testing will be essential to determine when CAFs are true therapeutic drivers and when they are markers of a broader resistant tumor ecosystem.
